# Number and nature of psychiatric emergency department visits in a tertiary hospital before, during, and after coronavirus pandemic

**DOI:** 10.3389/fpsyt.2024.1380401

**Published:** 2024-04-18

**Authors:** Claudia Aymerich, Borja Pedruzo, Gonzalo Salazar de Pablo, Nora Olazabal, Ana Catalan, Miguel Ángel González-Torres

**Affiliations:** ^1^ Psychiatry Department, Basurto University Hospital, Osakidetza, Basque Health Service, Bilbao, Spain; ^2^ Biobizkaia Health Research Institute, Organización Sanitaria Integrada (OSI) Bilbao-Basurto, Bilbao, Spain; ^3^ Centro de Investigación en Red de Salud Mental (CIBERSAM), Madrid, Spain; ^4^ Neuroscience Department, University of the Basque Country (UPV/EHU), Leioa, Spain; ^5^ Department of Child and Adolescent Psychiatry, Institute of Psychiatry, Psychology and Neuroscience, King’s College London, London, United Kingdom; ^6^ Early Psychosis: Interventions and Clinical-detection (EPIC) Lab, Department of Psychosis Studies, Institute of Psychiatry, Psychology and Neuroscience, King’s College London, London, United Kingdom; ^7^ Child and Adolescent Mental Health Services, South London and Maudsley, NHS Foundation Trust, London, United Kingdom; ^8^ Department of Child and Adolescent Psychiatry, Institute of Psychiatry and Mental Health, Hospital General Universitario Gregorio Marañón School of Medicine, Universidad Complutense, IiSGM, CIBERSAM, Madrid, Spain; ^9^ Department of Psychiatry, University of Oxford, Oxford, United Kingdom

**Keywords:** emergency, pandemic, coronavirus, psychiatric, ED

## Abstract

**Introduction:**

The COVID-19 pandemic has significantly impacted mental health globally, leading to a deterioration in the overall mental health of the population and changes across all healthcare levels, including emergency departments (ED). However, the evolution of the quantity and nature of psychiatric ED visits in the post-pandemic period remains uncertain.

**Aims:**

To examine changes in the number and nature of psychiatric emergencies at a general hospital before, during, and after the COVID-19 pandemic.

**Materials and methods:**

Psychiatric ED visits from a tertiary hospital in the Basque Country (Spain) between January 2019 and November 2023 were investigated. Electronical health registers detailing the number and nature of psychiatric care consultations were analyzed for the study timeframe. Three periods were then compared: pre-pandemic (from January 2019 to February 2020), pandemic (from March 2020 to January 2022), and post-pandemic (from February 2022 onwards).

**Results:**

16,969 psychiatric ED visits were recorded for the study period. The number of psychiatric ED visits remained stable from pre-pandemic (269.93 visits/month) to pandemic (264.48 visits/month) periods but experienced a significant rise during the post-pandemic period (330.00 visits/month; *t*=-6.42; p<0.001), which was not reflected in medical and traumatological visits. The proportion of visits for anxiety (Z=-2.97; p=0.003), suicidal ideation (Z=-5.48; p<0.001), and administrative and social consultations (Z=-5.69; p<0.001) increased over the course of the pandemic. In contrast, visits for schizophrenia and other psychotic disorders (Z=4.85; p<0.001), as well as unspecified behavioral alterations (Z=2.51; p=0.012), significantly decreased.

**Conclusion:**

The COVID-19 pandemic and its aftermath have altered the patterns of urgent psychiatric care, characterized by a sharp increase of average monthly number of consultations and a shift in their nature. Future efforts should focus on characterizing this surge in demand and enhancing both emergency services and outpatient settings.

## 1 Introduction

The global pandemic caused by the appearance of SARS CoV-2 in early 2020 (1) presented a challenge for the health systems worldwide, pushing most governments towards adopting confinements and other restrictive measures to preserve public health. The pandemic and its consequences not only strained the physical health of the population but also exacerbated mental health issues (2, 3). This was fueled by a context of uncertainty and cumulative macroeconomic losses worldwide (4, 5), along with social isolation and its secondary impact on mental health and physical inactivity during this period (6). Over the months following the onset of the spread of COVID-19, an increased incidence of mental health symptoms were noted among both clinical (7) and general (8, 9) population. A substantial increase in the burden of major depressive disorder and anxiety disorders was noted in 2020, with an increase of their prevalence of around 0.4% worldwide for both disorders (10) and a prevalence of suicidal ideation among general population of 12.1% (11), substantially higher than in studies prior to the pandemic.

Furthermore, the pandemic entailed the shutdown of schools, public entities, and even medical services to minimize the risk of exposure, slow the spread of the virus, and concentrate most available resources on the treatment of the COVID-19 disease. This, in turn, resulted in reduced or difficulted access to mental health treatment delaying or avoiding medical care for many people (12–14). In this context, emergency services became the primary and, for many patients, the only point of contact with mental health services, with a potential impact on help-seeking behavior. Therefore, a shift in the number and nature of emergency department (ED) visits was also expected.

Indeed, a decline in patient visits of up to 60% occurring after the onset on the pandemic was noted in general emergencies, both for adults and pediatric patients (15, 16). As for the number of psychiatric ED visits, an initial reduction was noted worldwide. In Western Australia, a 43% decrease in the number of total psychiatric ED visits was recorded over the last weeks of April 2020 compared to previous years (17). In Europe, a Portuguese hospital reported a 52% overall decrease during the same period, mostly at the expense of female patients presenting with mood disorders (18), while in Ireland a 21% decrease was noted between March and May (19), due to the lockdowns and fear of infection. On the other hand, a later increase in the number of ED visits was also expected once the most severe restrictive measures were lifted, due to the general worsening of population’s mental health and the overwhelm of outpatient services. There is evidence that the pandemic differentially affected not only diverse demographic groups, but also individuals with certain specific mental disorders compared to others (20). For instance, a large increase in the number of hospitalizations and care seeking behavior was found during the pandemic in patients with eating disorders (21) or personality disorders (22), among others. On the other hand, patients with obsessive-compulsive disorder did not show significant symptom deterioration during the pandemic (23). Therefore, changes in both the quantity and the reason for psychiatric ED visits was expected.

However, there is a shortage of research describing the evolution of psychiatric ED visits before, during and after the pandemic, not only in terms of numbers but also in the nature of consultations. To address this gap, we analyzed the electronic ED medical records from a tertiary hospital.

The primary objectives of this study were to (i) assess the evolution and presence of statistically significant variations in the monthly average number of visits to the psychiatric ED before, during, and after the COVID-19 pandemic; (ii) compare this evolution with that of medical and traumatological emergencies in the same periods; and (iii) assess the presence of changes in the nature of the psychiatric consultations before and after the pandemic. We hypothesized that ED visits for psychiatric care would decrease during the pandemic period, and then progressively increase in the following months and years to reach a higher number of ED visits compared to that prior to the COVID-19 pandemic. We also anticipated that this secondary increase in urgent care would be proportionally higher than that experienced by urgent care for other causes (medical and/or traumatological) in the same period.

## 2 Materials and methods

### 2.1 Population and data sources

This was a retrospective, observational study conducted at the Basurto University Hospital. This is a tertiary hospital located in the Basque Country in northern Spain, serving a population of 380,000 people. The Basurto University Hospital Research Ethics Committee gave ethical approval for the study (N.77.23 CEIHU). The present study followed the REporting of studies Conducted using Observational Routinely-collected Data (RECORD) guidelines for cohort studies (**Table S1**) (24).

We used an electronic health register [EHR; Osabide Global 2.0 (25)] to retrospectively identify all visits to the Basurto University Hospital’s Emergency Department over the course of four years (from 1^st^ of January 2019 to 30^th^ November 2023). In this register, ED visits are divided in several groups (medical, psychiatric, traumatological) by the ED staff at the time of their admission, according to the main reason of consultation.

The number of monthly visits and the main diagnosis resulting from each visit were obtained through the EHR. In this database, all patients are assigned a relevant ICD-10 (26) code by ED doctors at the time of their discharge. The data were de-identified to protect privacy and confidentiality of patients. To simplify the analysis, the primary diagnoses were classified into the following categories: neurodevelopmental disorders, schizophrenia spectrum and other psychotic disorders, depressive disorders, bipolar disorders, anxiety, obsessive-compulsive and related disorders, trauma- and stressor-related disorders, dissociative disorders, somatic symptom and related disorders, feeding and eating disorders, sleep-wake disorders, substance-related and addictive disorders, personality disorders, administrative and social consultations, behavioral alterations not otherwise specified, medical and traumatological consultations (where patients coming for a mainly medical or traumatological reason, but required specific advise by a psychiatry specialist for any reason), other consultations, and suicide and self-harm related consultations. This last category was then divided into three subgroups: suicide ideation/suicide risk, non-suicidal self-injuries, and suicide attempts. A detailed list of all the codes included under each category is available in **eMethods S1**. Drugs were divided into several subgroups as well: alcohol, stimulants, benzodiazepines and other hypnosedatives, opioids, cannabinoids, and unspecified/multiple substances (**eMethods S2**).

### 2.2 Statistical analyses

Three periods were defined for the study timeframe: pre-pandemic (from January 1^st^, 2019, to February 28^th^, 2020, marking the detection of the first COVID-19 case in Northern Spain), pandemic (from March 1^st^, 2020, to January 31^st^, 2022, when all pandemic-restrictions were lifted in Spain, including mandatory mask-wearing), and post-pandemic (from February 1^st^, 2022, to November 30^th^, 2023).

Monthly averages of psychiatric ED visits (in order to account for stational variations in the ED affluence) for each study period and category were calculated. After verifying the normality of the samples using Shapiro-Wilk test, a two-sample *t*-test with Bonferroni correction was applied to assess whether there were any significant differences between each pair of periods. The same procedure was followed for ED visits for medical care (as opposed to psychiatric care) for each study period.

Then, the nature of the psychiatric consultations and its variations over the study periods were assessed. First, the percentage of visits per diagnostic category was calculated for each study period, and pie charts were created to depict the results. Then, we evaluated the presence of statistically significant differences in the percentage of consultations per diagnostic category between the pre-pandemic and post-pandemic periods. As percentages were used, Z-Test for Proportions was used for those categories including more than 30 consultations per study period (27). The same procedure was followed to assess the presence of statistically significant differences among diagnostic subgroups.

R software, version 1.4.1106 (28) was used for all analyses. The significance level was set at p <0.05, two-sided.

## 3 Results

Throughout the study period, the hospital’s ED provided care for 16,969 individuals seeking psychiatric consultations and 450,105 individuals seeking medical or traumatological care. The number of monthly visit counts across the study period is available in **Table S2** and **Table S3**.

### 3.1 Average ED monthly visits

During the pre-pandemic period (from January 2019 to February 2020), 269.93 psychiatric visits/month were registered in the ED. In April 2020, shortly after the onset of the pandemic, 350 visits were registered, but over the following months the visits/month went down to the previous figures, averaging at 264.48 visits/month for the pandemic period. However, in the post-pandemic period, the number of consultations increased significantly, reaching 330.00 average visits/month. The pre-pandemic and pandemic periods did not significantly differ in average monthly visits (*t*=0.53; p=0.60). However, during the post-pandemic period a significantly higher number of monthly visits were recorded, compared to both the pre-pandemic (*t*=-7.29; p<0.001) and pandemic (*t*=-6.42; p<0.001) periods.

In contrast, medical and traumatological emergency visits displayed a distinct pattern, exhibiting a clinically and statistically significant decrease from the pre-pandemic period to the pandemic period (from 8155.71 visits/month to 6777.48 visits/month; *t*=4.92; p<0.001). In the post-pandemic period, the number of average monthly visits rose back to numbers similar to the pre-pandemic period (8183.77 visits/month). There were no statistically significant differences between the pre-pandemic and post-pandemic periods ED affluence (*t*=-0.20; p=0.84).

**Figure 1:**
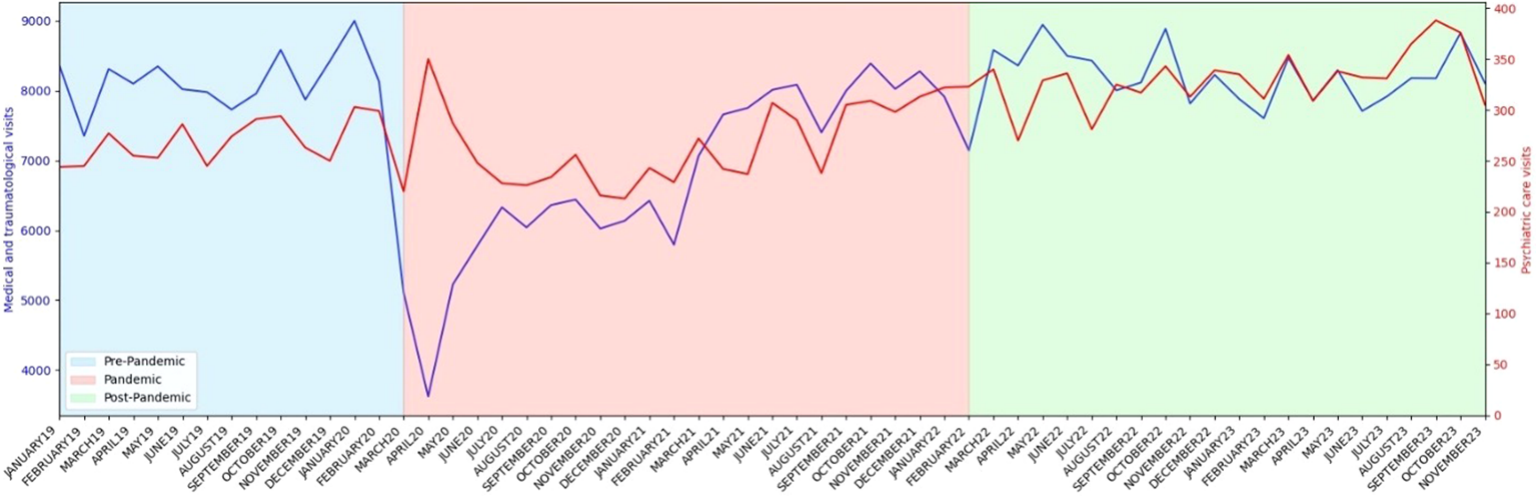
Absolute number of psychiatric care (in red) and medical and traumatological care (in blue) ED visits over the course of the study period.

In **Figure 1**, the evolution of the average monthly visits for psychiatric and medical/traumatological ED is detailed.

### 3.2 Changes in the nature of psychiatric ED visits

In the pre-pandemic period, the main reason for psychiatric ED visits were anxiety-related consultations (30.2%), followed by schizophrenia spectrum and other psychotic disorders (14.0%), substance-related and addictive disorders (9.7%), behavior alterations not otherwise specified in the discharge report (9.3%), and suicide and self-harm related consultations (8.1%). At that time, administrative and social consultations only represented 5.2% of the total.

**Figure 2:**
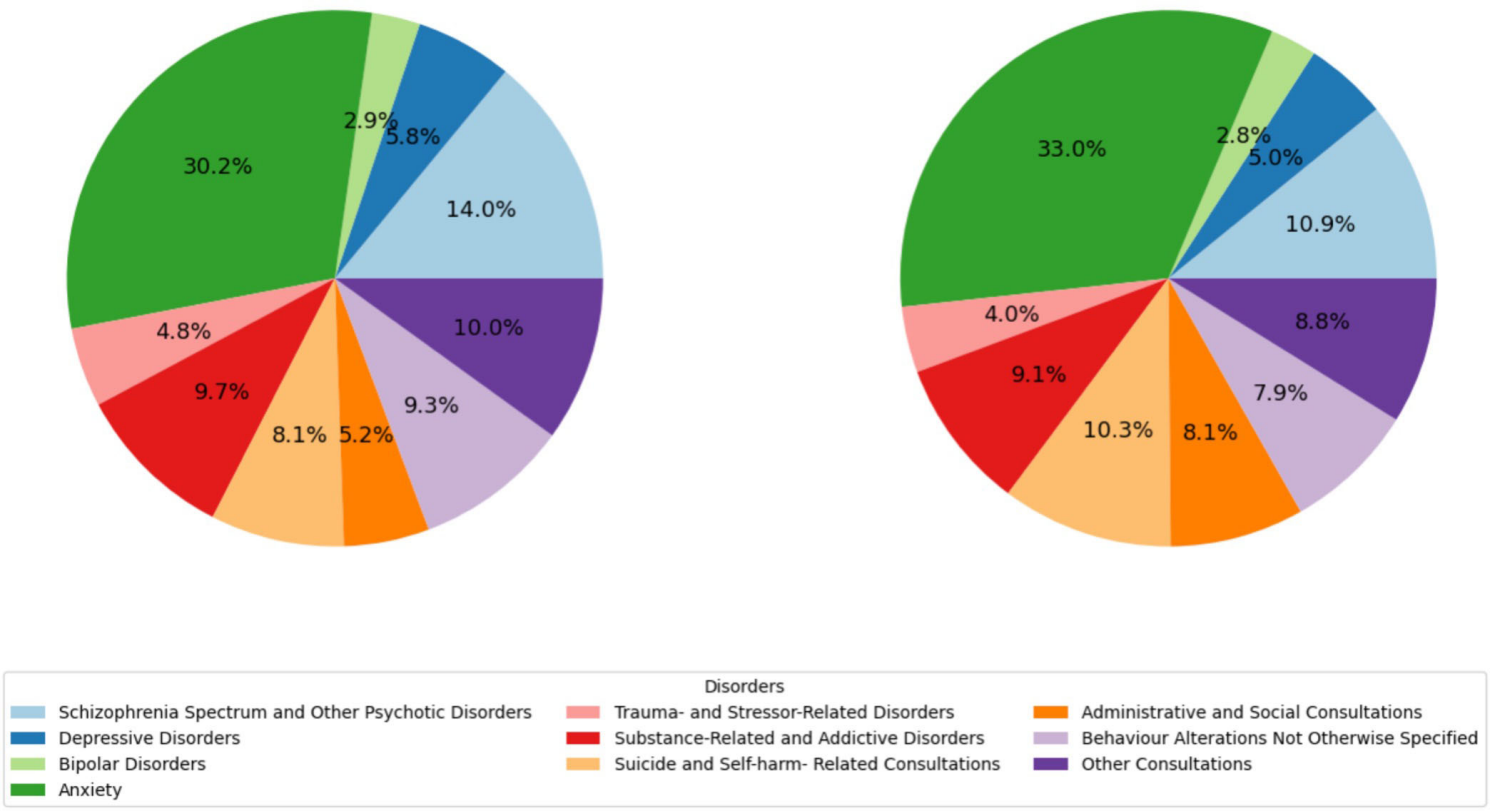
Reason for consultation of visits to the ED for psychiatric care in the pre-pandemic period (left) and the post-pandemic period (right).

During the post-pandemic period, however, some statistically significant changes in the characteristics of psychiatric ED visits were noted. Although the absolute number of visits for almost all the diagnostic categories grew (**Table 1**), anxiety-related consultations rose to 33.0% of the total (Z=-2.97; p=0.003). Suicide and self-harm related consultations also experienced a notable growth (10.3%; Z=-3.83; p<0.001), along with administrative and social consultations (8.1%; Z=-5.69; p<0.001). On the other hand, a statistically significant reduction was detected in the percentages of consultations for schizophrenia spectrum and other psychotic disorders (10.9%; Z=4.85; p<0.001), trauma- and stressor-related disorders (from 4.8% to 4.0%; Z=1.99; p=0.047), behavior alterations not otherwise specified (7.9%; Z=2.51; p=0.012), and liaison psychiatry consultations in patients with a primary medical and/or traumatological diagnosis (from 3.94% to 2.95%; Z=2.76; p=0.006). The percentage of visits for depressive disorders and bipolar affective disorders, sleep-wake disorders, substance-related and addictive disorders, and personality disorders, did not show significant changes between the two periods (**Figure 2**, **Table 2**).

**Table 1:** Average monthly visit counts by diagnostic category for psychiatric care patients across different study periods.

	Average monthly visit counts		
	Pre-pandemic	Pandemic	Post-pandemic
Neurodevelopmental Disorders	1.36	1.65	1.68
Schizophrenia Spectrum and Other Psychotic Disorders	37.86	33.30	35.45
Depressive Disorders	15.79	12.87	16.36
Bipolar Disorders	7.86	9.26	9.14
Anxiety	81.64	81.00	107.82
Obsessive-Compulsive and Related Disorders	1.21	0.52	1.18
Trauma- and Stressor-Related Disorders	12.93	9.96	13.00
Dissociative Disorders	0.50	0.61	1.64
Somatic Symptom and Related Disorders	0.50	0.30	0.32
Feeding and Eating Disorders	0.43	2.09	1.86
Sleep-Wake Disorders	3.21	2.48	4.00
Substance-Related and Addictive Disorders	26.07	22.30	29.77
Personality Disorders	4.50	5.43	4.09
Suicide and Self-harm- Related Consultations	21.79	23.13	33.73
* Suicidal ideation/Suicidal risk*	*5.93*	*9.13*	*13.95*
* Non-Suicidal Self Injuries*	*2.93*	*2.83*	*3.82*
* Suicide attempts*	*12.93*	*11.17*	*15.95*
Administrative and Social Consultations	13.93	18.17	26.41
Behavior Alterations Not Otherwise Specified	25.21	25.48	25.91
Medical and Traumatological Consultations (Psychiatry Liaison Service)	10.64	8.57	9.64
Other Consultations	2.50	2.74	2.73
**Total monthly visit counts**	**269.93**	**264.48**	**330.00**

Bold letters are used to indicate it is the sum of all the above values (total monthly visit counts).

**Table 2:** Consultation reason distribution in psychiatric emergency department visits pre-pandemic period (left) *vs*. post-pandemic period (right).

Category	Pre-Pandemic (%)	Post-Pandemic (%)	Z-Test	P value
Schizophrenia Spectrum and Other Psychotic Disorders	14.02%	10.86%	4.85	<0.001*
Depressive Disorders	5.85%	5.01%	1.83	0.067
Bipolar Disorders	2.91%	2.80%	0.33	0.738
Anxiety	30.24%	33.03%	-2.97	0.003*
Trauma- and Stressor-Related Disorders	4.79%	3.98%	1.99	0.047*
Sleep-Wake Disorders	1.19%	1.22%	-0.16	0.874
Substance-Related and Addictive Disorders	9.66%	9.12%	0.92	0.358
Personality Disorders	1.67%	1.25%	1.75	0.079
Suicide and Self-Harm Related Consultations	8.07%	10.33%	-3.83	<0.001*
* Suicidal ideation/Suicidal risk*	*2.20%*	*4.23%*	-5.48	<0.001*
* Non-Suicidal Self Injuries*	*1.08%*	*1.16%*	-0.38	0.706
* Suicide attempts*	*4.79%*	*4.83%*	-0.09	0.926
Administrative and Social Consultations	5.16%	8.09%	-5.69	<0.001*
Behavior Alterations Not Otherwise Specified	9.34%	7.94%	2.51	0.012*
Medical and Traumatological Consultations (Psychiatry Liaison Service)	3.94%	2.95%	2.76	0.006*

* indicates statistical significance.

Subgroup analysis also revealed important findings. When suicide and self-harm related consultations were divided into three subgroups, no statistically significant changes were appreciated in the percentage of consultations for non-suicidal self-injuries (from 1.08% to 1.16%; Z=-0.38; p=0.706) nor suicide attempts (from 4.79% to 4.83%; Z=-0.09; p=0.926). However, visits for suicidal ideation or suicidal risk significantly increased from 2.20% to 4.23% (Z=-5.48; p<0.001*), which suggests that the relative increase of the whole category is at the expense of this subgroup of visits.

On the other hand, no significant changes were noted between the two periods in terms of the substance that caused the substance-related and addictive disorders consultations, with alcohol representing well over 50% of those visits for both periods (**Table S3**).

## 4 Discussion

To the best of the authors’ knowledge, this is the first study to comprehensively investigate the evolution in the number and nature of psychiatric emergencies at a general hospital before, during, and after the coronavirus pandemic.

Our findings show that the frequency of psychiatric emergencies in the ED remained consistent during the pandemic, not deviating significantly from the affluency observed in the previous year. This finding stands in contrast to other research, which has reported both and increase (29) and decrease (30–32) in such emergencies. The variance seen in these studies could be attributed to differences in the pandemic’s impact across various countries and in healthcare management practices (33, 34). For instance, previous studies in this area report a differential impact of the pandemic on primary care utilization for mental disorders across European countries, highly correlating with containment strategies used in each of them (35). Furthermore, it’s worth noting that most studies focused solely on the year 2020, not accounting for the continued pandemic-related restrictions like lockdowns, social distancing, and the reduction in outpatient medical services that persisted beyond this year.

However, in the period following the lifting of SARS-COV-2 related restrictions (post-pandemic period in our study), our research detected a notable rise in the number of psychiatric emergencies, that has persisted without any indication of diminishing. This surge was not mirrored in the total number of ED consultations for medical care, which decreased during 2020 and subsequently reverted to pre-pandemic figures.

There are several possible explanations for this increase in psychiatric ED visits after the initial waves of the pandemic. There is consistent evidence indicating changes in the levels of psychological distress in the population during the pandemic, characterized by cyclical periods of deterioration (often coinciding with new COVID-19 waves and the implementation of new restrictive measures) followed by recovery. However, data suggest that this recovery did not reach pre-pandemic levels, with a slow, steady deterioration of the overall mental health of the population as the pandemic progressed (36, 37), often referred to as the ‘second pandemic’ (38, 39). The health crisis and subsequent lockdowns have been characterized by unpredictability and uncertainty, with numerous restrictions, an increase in unemployment rates, and changes in living standards. These factors have posed sustained stress for many individuals, including those with pre-existing mental health diagnoses and others who had not previously required psychiatric care. Economic recession and social isolation are known factors that exacerbate rates of psychopathology, suicidal behaviors, and substance use, among others, as has previously been reported in other major health crisis (40). Furthermore, these changes have hindered access to mental health services, which were already precarious and overwhelmed in many communities (41, 42). Numerous countries implemented alternatives to address these issues, prominently telemedicine and hotline services. However, the preference of patients is still face to face therapy, and these measures have often proven insufficient (43). Almost two thirds of mental health professionals expressed negative thoughts on the efficacy of telemental health, prominently technology problems and concerns on its safety in certain settings and disorders (44).

Another important finding of our research is that a significant shift has been found in the nature of ED visits after the pandemic. When compared to the pre-pandemic period, there is a substantial increase in consultations related to anxiety, suicidal attempts, and self-harm behaviors. The rising suicide attempt rates during this period is as concerning as consistent with reports from other settings, such as inpatient units (45). As of now, suicide ranks as the leading cause of non-natural death in Spain among those aged 15 to 29 (46), and in 2020, it led to a greater loss of potential life years than the coronavirus itself (47). The results of our study suggest that this trend likely persisted in the years after the pandemic, in line with initial predictions made at the start of the pandemic (48) and similar findings in other countries (49, 50). Recent reports suggest this increase in suicidality to be mainly driven by suicides in older adults, potentially impacted by the isolation, the loss, and bereavement they have faced in the context of particularly high mortality rates in Spain (51). There is an urgent need for the implementation of strategies targeted to particularly vulnerable populations to address and alleviate this critical global health issue. It’s also noteworthy the rise in administrative and social consultations in recent years. These consultations encompass a range of issues, including requests for overnight stays, medical reports or prescriptions, and other administrative matters. The pandemic and its consequences have significantly impacted Spain and its population’s economic conditions (5), with a national health system that was not prepared for the burden of the pandemic due to insufficient funding after budget cuts in the previous economic crisis, that left the country with lower social and health expenditure per capita than the European Union average (52). This, added to the overburdening of primary overburdening of primary care services (53) and a decrease and delay of face-to-face assessments, resulted in patients having increasingly directed these types of non-urgent inquiries and procedures to the ED.

Conversely, there has been a notable decrease in the percentage of urgent care visits for schizophrenia and other psychotic disorders. Additionally, there’s been a reduction in the proportion of urgent consultations related to behavioral alterations not otherwise specified in discharge summaries, many of which are associated with psychotic disorders. This might be reflecting two opposite (but possibly complementing) realities. On the one hand, a prior study in the same population suggested that, compared to individuals with other severe mental disorders, patients with schizophrenia did not experience a significant decline in mental health during and after the COVID-19 pandemic (20). Moreover, patients with schizophrenia maintained adherence to their treatment throughout this period (54). On the other hand, this decrease in the urgent care visits could reflect a loss of follow-up of this group of patients, an especially vulnerable subpopulation that were also at increased risk of COVID-19 hospitalization and mortality risk (55).

This observed shift in urgent care demand patterns post-pandemic likely mirrors a sustained worsening in the mental health of the population, a challenge that outpatient settings may be struggling to meet due to overwhelming demand. This trend, far from moderating, seems to be escalating, as evidenced by historically high levels of urgent psychiatric care at the time of writing this article. This situation urgently calls for not only strengthening emergency services but also enhancing outpatient settings, which are ideally suited for managing most of these demands (56). Resources allocation is highly challenging in a pandemic, where emergency relief resources are needed simultaneously at multiple affected areas (57). However, this pandemic poses an opportunity to prepare for future health crisis and disasters. Additionally, there is a need for developing preventive and social strategies to mitigate the socio-economic impacts of the pandemic. It’s also crucial to acknowledge the potential impact of this increased care demand on healthcare workers, a group significantly affected by the SARS-COV-2 pandemic (58), and to provide additional training to primary care professionals to help them deal with psychological crisis outside the ED (59). Finally, basic self-care strategies can be taught to population to help them recognize and deal with signs of distress, along with education on the use of urgent care settings.

The findings of this study should be interpreted within the context of several limitations. First, the study is subject to the inherent limitations of observational, retrospective designs, where establishing causality between the examined factors is challenging. Nonetheless, the multifaceted impact (health-related (60, 61), social (62, 63), and economic (5), among others) of the COVID-19 pandemic on the mental health of the population has been extensively demonstrated. Second, the limited range of study variables, restricting the investigation to important factors such as the patients’ sex or age. Third, the coding of discharge diagnoses in an emergency department is primarily intended for clinical purposes and is often made under time-sensitive circumstances. Therefore, syndromic diagnoses (such as anxiety) coexist with more specific ones like schizophrenia. And fourth, only the primary coding of each consultation was considered in the analysis. Consequently, it is possible that secondary diagnoses may have been overlooked.

On the other hand, while demand patterns for psychiatric assistance encompass economic and cultural factors (64), Spain’s provision of free and universal healthcare indicates that the data collected in this study likely offers a realistic perspective, unaffected by economic constraints. Furthermore, this study was conducted in a hospital where the psychiatry department serves as a primary point of contact for urgent care, and all patients included were assessed by trained psychiatrists. This contrasts with other centers where general physicians initially manage all urgent consultations, escalating to specialists only in certain cases. Consequently, a vast majority of psychiatric consultations, irrespective of their level of complexity or severity, have been included in this study. This approach significantly enhances the internal validity of the data.

Our findings revealed that COVID-19 pandemic and its aftermath have profoundly altered urgent psychiatric care. Future research should aim to characterize how these past few years have impacted particularly vulnerable subpopulations such as adolescents (65), women (66), and those economically hard-hit by the pandemic (67, 68), who seem more susceptible to psychopathological decline, while other populations might have maintained adequate mental health levels throughout the pandemic. What remains unanswered, however, is whether this has led to an increased demand in any of these subgroups, or if, on the contrary, they remain unable to even access urgent care services.

## 5 Conclusions

Our study revealed a disproportionate rise in the number of psychiatric consultations in the emergency department during the post-pandemic period, relative to both the pre-pandemic and pandemic periods, with a notable increase in consultations related to anxiety, suicidal ideation, and self-harm, as well as those linked to administrative and social issues, possibly in relation to the negative impact of the pandemic on Spain’s economy. Conversely, there was a relative decrease in the number of patients presenting with schizophrenia and other psychotic disorders during the same timeframe, which could reflect both a better adaptation to confinement and isolation measures and a loss of follow-up of particularly vulnerable patients. Health systems and particularly urgent care settings should prepare for potential upcoming health crisis, with sufficient budget, resource allocation, and preventive measures. Health systems and particularly urgent care settings should prepare for potential upcoming health crisis, with sufficient budget, resource allocation, and preventive measures.

## Data availability statement

The raw data supporting the conclusions of this article will be made available by the authors, without undue reservation.

## Ethics statement

The Basurto University Hospital Research Ethics Committee gave ethical approval for the 106 study (N.77.23 CEIHU).

## Author contributions

CA: Conceptualization, Data curation, Methodology, Writing – original draft, Writing – review & editing. BP: Data curation, Validation, Writing – original draft, Writing – review & editing. GS: Conceptualization, Methodology, Writing – original draft, Writing – review & editing. NO: Data curation, Formal analysis, Writing – original draft, Writing – review & editing. AC: Supervision, Validation, Writing – original draft, Writing – review & editing. MG: Supervision, Validation, Writing – original draft, Writing – review & editing.
