# Supplementary material for: Number and nature of psychiatric emergency department visits in a tertiary hospital before, during, and after coronavirus pandemic
Source: Front Psychiatry. 2024 Apr 18;15:1380401. doi: 10.3389/fpsyt.2024.1380401 (PMC11063275; doi:10.3389/fpsyt.2024.1380401)
Supplement: Supplementary file 1 [file Table_1.docx]

Supplementary Material

**Table S1**: The RECORD statement – checklist of items, extended from the STROBE statement, that should be reported in observational studies using routinely collected health data…………..Pages 1-8

**eMethods S1**: Diagnostic codes included under each category…………….………………Pages 9-18

**eMethods S2**: Substances included under each category..……………………..………………Page 19

**Table S2:** Number of monthly visit counts for psychiatric care patients across the study period…….. …………………………………………………………………………………..………………Page 20

**Table S3:** Number of monthly visit counts for medical and traumatological care patients across the study period.…………………………………………………………………………………….Page 21

**Table S4:**. Number of monthly visits and percentage of the monthly total per substance over the study period….………………………………………………………………………………….Page 22

# **Table S1:** The RECORD statement – checklist of items, extended from the STROBE statement, that should be reported in observational studies using routinely collected health data

|  | **Item No.** | **STROBE items** | **Location in manuscript where items are reported** | **RECORD items** | **Location in manuscript**  **where items are reported** |
| --- | --- | --- | --- | --- | --- |
| **Title and abstract** | | | | | |
|  | 1 | (a) Indicate the study’s design with a commonly used term in the title or the abstract (b) Provide in the abstract an informative and balanced  summary of what was done and what was found | Title | RECORD 1.1: The type of data used should be specified in the title or  abstract. When possible, the name of the databases used should be included.  RECORD 1.2: If applicable, the geographic region and timeframe within which the study took place should be reported in the title or  abstract.  RECORD 1.3: If linkage between databases was conducted for the study, this should be clearly stated in the title or abstract. | Title |
| **Introduction** | | | | | |
| Background rationale | 2 | Explain the scientific background and rationale for the investigation being reported | Introduction |  | Introduction |
| Objectives | 3 | State specific objectives, including any prespecified  hypotheses | Introduction |  | Introduction |
| **Methods** | | | | | |
| Study Design | 4 | Present key elements of study design early in the paper | Methods |  | Methods |
| Setting | 5 | Describe the setting, locations, and relevant dates, including  periods of recruitment, exposure, follow-up, and data collection | Methods |  | Methods |
| Participants | 6 | *(a) Cohort study* - Give the  eligibility criteria, and the  sources and methods of selection of participants. Describe methods of follow-up  *Case-control study* - Give the eligibility criteria, and the  sources and methods of case ascertainment and control  selection. Give the rationale for the choice of cases and controls *Cross-sectional study* - Give the eligibility criteria, and the  sources and methods of selection of participants  *(b) Cohort study* - For matched studies, give matching criteria and number of exposed and unexposed  *Case-control study* - For matched studies, give matching criteria and the number of  controls per case | Methods | RECORD 6.1: The methods of study  population selection (such as codes or algorithms used to identify subjects) should be listed in detail. If this is not possible, an explanation should be provided.  RECORD 6.2: Any validation studies of the codes or algorithms used to  select the population should be referenced. If validation was conducted for this study and not published  elsewhere, detailed methods and results should be provided.  RECORD 6.3: If the study involved linkage of databases, consider use of a flow diagram or other graphical display to demonstrate the data linkage process, including the number of individuals with linked data at each  stage. | Methods |
| Variables | 7 | Clearly define all outcomes,  exposures, predictors, potential confounders, and effect modifiers. Give diagnostic  criteria, if applicable. |  | RECORD 7.1: A complete list of codes and algorithms used to classify  exposures, outcomes, confounders, and effect modifiers should be provided. If these cannot be reported, an  explanation should be provided. | Methods |
| Data sources/ measurement | 8 | For each variable of interest, give sources of data and details of methods of assessment (measurement).  Describe comparability of  assessment methods if there is more than one group | Methods |  | Methods |
| Bias | 9 | Describe any efforts to address  potential sources of bias | Methods |  | Methods |
| Study size | 10 | Explain how the study size was  arrived at | Methods |  | Methods |
| Quantitative variables | 11 | Explain how quantitative variables were handled in the  analyses. If applicable, describe which groupings were chosen, and why | Methods |  | Methods |
| Statistical methods | 12 | 1. Describe all statistical methods, including those used to control for confounding 2. Describe any methods used to examine subgroups and interactions 3. Explain how missing data were addressed 4. *Cohort study* - If applicable, explain how loss to follow-up was addressed   *Case-control study* - If applicable, explain how  matching of cases and controls was addressed  *Cross-sectional study* - If  applicable, describe analytical methods taking account of  sampling strategy   1. Describe any sensitivity analyses | Methods |  | Methods |
| Data access and  cleaning methods |  | .. |  | RECORD 12.1: Authors should  describe the extent to which the investigators had access to the database population used to create the study population.  RECORD 12.2: Authors should provide information on the data  cleaning methods used in the study. | Methods |
| Linkage |  | .. |  | RECORD 12.3: State whether the |  |
|  |  |  |  | study included person-level, institutional-level, or other data linkage across two or more databases. The methods of linkage and methods of linkage quality evaluation should be  provided. | Methods |
| **Results** |  |  |  |  |  |
| Participants | 13 | 1. Report the numbers of individuals at each stage of the study (*e.g.*, numbers potentially   eligible, examined for eligibility, confirmed eligible, included in the study, completing follow-up, and analysed)   1. Give reasons for non- participation at each stage. 2. Consider use of a flow diagram | Results | RECORD 13.1: Describe in detail the selection of the persons included in the study (*i.e.,* study population selection) including filtering based on data quality, data availability and linkage. The selection of included persons can be described in the text and/or by means of the study flow diagram. | Results |
| Descriptive data | 14 | 1. Give characteristics of study participants (*e.g.*, demographic, clinical, social) and information on exposures and potential   confounders   1. Indicate the number of participants with missing data for each variable of interest 2. *Cohort study* - summarise follow-up time (*e.g.*, average and total amount) | Results |  | Results |
| Outcome data | 15 | *Cohort study* - Report numbers  of outcome events or summary measures over time  *Case-control study* - Report numbers in each exposure  category, or summary measures of exposure  *Cross-sectional study* - Report numbers of outcome events or  summary measures | Results |  | Results |
| Main results | 16 | 1. Give unadjusted estimates and, if applicable, confounder- adjusted estimates and their precision   (e.g., 95% confidence interval). Make clear which  confounders were adjusted for and why they were included   1. Report category boundaries when continuous variables were categorized 2. If relevant, consider translating estimates of relative risk into absolute risk for a meaningful time period | Results |  | Results |
| Other analyses | 17 | Report other analyses done—e.g., analyses of  subgroups and interactions, and sensitivity analyses | Results |  | Results |
| **Discussion** | | | | | |
| Key results | 18 | Summarize key results with  reference to study objectives | Discussion |  | Discussion |
| Limitations | 19 | Discuss limitations of the study, taking into account sources of potential bias or imprecision.  Discuss both direction and magnitude of any potential bias | Discussion | RECORD 19.1: Discuss the implications of using data that were not created or collected to answer the  specific research question(s). Include discussion of misclassification bias, unmeasured confounding, missing data, and changing eligibility over time, as they pertain to the study being reported. | Discussion |
| Interpretation | 20 | Give a cautious overall interpretation of results considering objectives,  limitations, multiplicity of  analyses, results from similar studies, and other relevant  evidence | Discussion |  | Discussion |
| Generalisability | 21 | Discuss the generalisability (external validity) of the study  results | Discussion |  | Discussion |
| **Other Information** |  |  |  |  |  |
| Funding | 22 | Give the source of funding and the role of the funders for the present study and, if applicable, for the original study on which  the present article is based | Funding |  | Funding |
| Accessibility of protocol, raw data, and programming  code |  | .. | Data availability disclosure | RECORD 22.1: Authors should provide information on how to access the supplemental information such as the study protocol, raw data, or  programming code. | Data availability disclosure |

*Reference: Benchimol EI, Smeeth L, Guttmann A, Harron K, Moher D, Petersen I, Sørensen HT, von Elm E, Langan SM, the RECORD Working Committee. The REporting of studies Conducted using Observational Routinely-collected health Data (RECORD) Statement. *PLoS Medicine* 2015.

**eMethods S1**: Diagnoses included under each category.

| **Neurodevelopmental Disorders** |
| --- |
| Aggressiveness in Autism Spectrum Disorder  Agitation in Intellectual Disability  ASD  Asperger Syndrome  Autism  Autism Spectrum Disorder  Behavioural Alteration in Intellectual Disability  Behavioural Alteration in Pervasive Developmental Disorder  Behavioural Change in Autism Spectrum Disorder  Behavioural Change in Pervasive Developmental Disorder  Behavioural Change in the Context of Intellectual Disability  Behavioural Change. Autism Spectrum Disorder  Borderline Intellectual Functioning  Childhood Autism – Active State  Generalized Developmental Disorder  Intellectual Disability  Pervasive Developmental Disorder  Tourette Syndrome |
| **Schizophrenia Spectrum and Other Psychotic Disorders** |
| Acute Psychotic Relapse  Agitation in Schizophrenia  Anxiety in Schizophrenia  Anxiety in the Context of a Psychotic Disorder  Auditory Hallucinations  Brief and Limited Psychotic Episode  Brief Psychotic Disorder  Chronic Psychosis  Chronic Schizophrenia  Decompensated Schizophrenia  Delusion/Paranoia  Delusional Disorder  Delusional Ideas  Delusional Ideas Disorder  Delusional Ideation  Ekbom’s Syndrome  First Psychotic Episode  Hallucinatory Syndrome  Non-Specified Psychosis  Othello Syndrome  Paranoid Schizophrenia  Paranoid State  Possible Psychotic Decompensation  Possible Psychotic Episode Under Study  Postpartum Psychosis  Probable Psychotic Decompensation  Psychosis  Psychosis and Stimulant Use  Psychosis Under Study  Psychotic Episode  Psychotic Reaction  Psychotic Relapse  Psychotic Symptoms  Psychotic Symptoms and THC Use  Psychotic Symptoms Under Study  Residual Schizophrenia  Resistant Schizophrenia  Schizoaffective Disorder  Schizoaffective Relapse  Schizophreniform Disorder  Schizophreniform Psychotic Disorder  Sensoperceptive Alterations  Simple Schizophrenia  Substance-Induced Psychotic Disorder  Undifferentiated Schizophrenia  Unspecified Psychotic Disorder |
| **Depressive Disorders** |
| Agitated Depression  Agitation in the Context of a Depressive Episode  Anxiety in Resistant Depression  Anxiety in the Context of Resistant Depression  Anxious Depression  Depression  Depression with Psychotic Symptoms  Depressive Disorder  Depressive Disorder with Obsessive Components  Depressive Episode  Depressive Episode in the Context of Neurological Disease  Depressive Episode with Psychotic Symptoms  Depressive Symptoms  Depressive Syndrome  Dysthymia  Hypothymia  Major Depression  Major Depression with Psychotic Symptoms  Major Depressive Disorder, Relapse  Major Depressive Episode  Major Depressive Syndrome with Several Previous Admissions  Mild Depressive Episode  Moderate Depressive Episode  Mood Dysregulation Disorder  Possible Depressive Episode Depressive Relapse  Postpartum Depression  Recurrent Depressive Disorder  Recurrent Depressive Disorder  Recurrent Depressive Disorder, Current Episode Severe  Sadness  Severe Depressive Episode  Treatment-Resistant Depression  Unspecified Depressive Disorder  Unspecified Depressive Syndrome |
| **Bipolar Affective Disorder** |
| Affective Decompensation in Bipolar Affective Disorder  BD, Depressive Episode  BD, Manic Episode  BD, Mixed Episode  BD, Type II  Bipolar  Bipolar Affective Disorder  Bipolar Affective Disorder, Depressive Episode  Bipolar Affective Disorder, Manic Episode  Bipolar Affective Disorder, Mixed Episode  Bipolar Depression  Bipolar Disorder I  Bipolar Disorder Type I  Depression with Mixed Features  Hypomania  Hypomanic Episode  Mania  Manic Decompensation  Manic Episode  Manic Episode with Psychotic Symptoms  Mixed Decompensation  Mixed Episode  Mixed State  Possible Manic Episode  Possible Mixed Episode  Possible Mixed Symptoms |
| **Anxiety** |
| Anguish Crisis  Anxiety  Anxiety and Behavioural Alteration  Anxiety Crisis  Anxiety Disorder  Anxiety Disorder Possibly Related to Psychopharmacology  Anxiety Episode  Anxiety in Mood Disorder  Anxiety Under Study  Anxiety vs Dizziness  Distress  Dyspnoea Related to Anxiety  Generalized Anxiety Disorder  Generalized Anxiety Syndrome  Impulse Phobias  Nervousness  Non-Specified Anxiety Disorder  Other Anxiety Disorders  Panic Disorder  Panic Disorder with Agoraphobia  Phobia  Probable State of Anxiety  Psychomotor Restlessness  Reports Anxiety  Restlessness  Restlessness and Insomnia  Social Phobia  Specific Phobia  State of Anxiety  Unspecified Anxiety Disorder |
| **Obsessive-Compulsive and Related Disorders** |
| Obsessive Disorder Under Study  Obsessive Ideation  Obsessive Symptoms  Obsessive Syndrome  Obsessive-Compulsive Disorder  OCD  Severe Obsessive-Compulsive Disorder |
| **Trauma- and Stress-Related Disorders** |
| Acute Stress  Acute Stress Reaction  Acute Stress Reaction  Adaptive Disorder  Adaptive Disorder with Mixed Symptoms  Adaptive Reaction  Adaptive Reaction  Adaptive Reaction (Grief)  Adaptive Reaction with Anxiety  Adaptive Reaction with Anxiety and Depression  Adaptive Reaction with Anxious Mood  Adaptive Reaction with Mixed Emotions  Adaptive Symptoms  Adjustment Disorder  Adjustment Disorder with Anxiety  Adjustment Disorder with Anxiety and Depression  Adjustment Disorder with Behavioural Alteration  Adjustment Disorder with Depressive Mood  Adjustment Disorder with Depressive Mood and Anxiety  Adjustment Disorder with Mixed Symptoms  Adjustment Disorder with Predominant Anxiety  Anxiety Reaction  Anxious-Depressive Adjustment Disorder  Attachment Disorder  Bereavement  Depressive Adjustment Disorder  Emotional Overwhelm  Long-Tern Adjustment Disorder  Normal Bereavement  Post-Traumatic Stress Disorder  Post-Traumatic Symptoms  PTSD  Reactive Anxiety  Unresolved Grief |
| **Dissociative Symptoms and Disorders** |
| Derealization / Depersonalization  Dissociative Amnesia  Dissociative Disorder  Dissociative Episode  Dissociative State  Non-Specified Dissociative Disorder  Probable Dissociative Episode  Psychogenic Amnesia  Remitted Dissociative Disorder |
| **Somatic Symptoms and Related Disorders** |
| Conversion Disorder  Conversive Crisis  Conversive Disorder  Conversive Episode  Possible Somatoform Disorder  Pseudocrisis  Pseudoseizure  Somatic Symptom Disorder  Somatoform Disorder |
| **Feeding and Eating Disorders** |
| Anorexia  Anorexia Nervosa  Bulimia Nervosa  Eating Disorder |
| **Sleep-Wake Disorders** |
| Insomnia  Insomnia Without Current Evidence of a Neurological Disease to Justify It |
| **Substance-Related and Addictive Disorders** |
| Acute Alcoholic Intoxication  Admission for Substance Cessation  Aggressiveness. Alcohol and Cocaine Use  Agitation in the Context of Acute Alcohol Intoxication  Alcohol Abuse  Alcohol Abuse and Probable Benzodiazepine Abuse  Alcohol Addiction  Alcohol Dependence  Alcohol Dependence with Alcohol-Induced Mood Disorder  Alcohol Ingestion  Alcohol Intoxication Acute Alcohol Intoxication  Alcohol Use  Alcohol Withdrawal  Alcoholic hallucinosis  Alcoholic Ketoacidosis  Alcoholism  Amphetamine Abuse  Amphetamine Dependence  Amphetamine Intoxication  Amphetamine Use  Anxiety and Alcohol Intoxication  Behavioural Alteration due to Alcohol and Cocaine Use  Behavioural Alteration due to Substance Use  Behavioural Alteration in Alcohol Use Context  Behavioural Alteration in Substance Intoxication Context  Behavioural Alteration Related to Amphetamine Use  Benzodiazepine Abuse  Benzodiazepine Dependence  Benzodiazepine Use  Benzodiazepine Withdrawal  BZD Abuse  BZD Dependence  Cannabinoid Dependence  Cannabis Intoxication  Cannabis Use  Chronic Alcoholism  Cocaine Abuse  Cocaine Use  Confusional Clinic / Toxic Psychosis  Consumption of Alcohol and THC  Dependence on Alcohol  Dependence on Other Psychoactive Substance  Drug Abuse  Drug Addiction  Drug Dependence  Enolic Intoxication  Fall in Probable Relation to Alcohol Dependence Syndrome  Harmful Alcohol Use  Harmful Cocaine Use  Harmful Use of Alcohol and Benzodiazepines  Harmful Use of Substances  Hypnosedative Dependence  Hypnosedative Use  Lisdexamfetamine Abuse  Medication Abuse  Methadone Withdrawal  Multiple Substance Abuse  Multiple Substance Dependence  Opioid Abuse  Opioid Dependence  Opioid Withdrawal  Pregabalin Abuse  Psychomotor Agitation and Substance Use  Psychotropic Drug Abuse  Solvent Abuse  Speed Use  Stimulant Abuse  Stimulant Dependence  Stimulant Use  Stimulant Use  Substance Abuse  Substance Dependence  Substance Dependence  Substance Use  Substance Use in Bipolar Disorder  Substance Use Under Study  Substance Withdrawal  THC Use  THC Withdrawal  Toxic Substance Use  Withdrawal |
| **Personality Disorders** |
| Antisocial Personality Disorder  Behavioural Alteration in Personality Disorder  Borderline Disorder  Borderline Personality  Borderline Personality Disorder  Deferred Diagnosis in Axis II  Dissocial Personality  Dissocial Personality Disorder  Emotional Instability Disorder  Emotional Instability Personality Disorder  Histrionic Personality Disorder  Mixed Personality Disorder  Paranoid Personality Disorder  Personality Disorder  Unspecified Personality and Adult Behaviour Disorder  Unspecified Personality Disorder |
| **Suicide and Self-Harm Related Consultations** |
| Suicidal Ideation / Suicide Risk  Autolytic Ideation  Crisis with Suicidal Risk  Ideation of Death  Suicidal Ideation  Non-Suicidal Self-Injury  Chest Self-Inflicted Wounds  Forearm Self-Inflicted Wounds  Left Forearm Self-Inflicted Wounds  Neck Self-Inflicted Wounds  Self-Harm  Self-Harming Gesture  Self-Inflicted Wounds  Self-Injuries  Self-Injury  Suicide Attempt  Alcohol and Medication Ingestion with Suicidal Intent  Autolysis  Autolysis with Alcohol and BZD Use  Autolytic Attempt with Paracetamol  Autolytic Gesture  BZD Ingestion with Suicidal Ideation  Intoxication by Tetracyclic Antidepressants  Intoxication with Suicidal Intent  Non-Accidental Medication Ingestion  Parasuicidal Gesture  Severe BZD Intoxication  Suicide Attempt  Voluntary Antipsychotic Intoxication  Voluntary Bleach Ingestion  Voluntary BZD Ingestion with Suicidal Intent  Voluntary Lamotrigine Intoxication  Voluntary Medication and Alcohol Intoxication  Voluntary Medication Ingestion  Voluntary Soap Ingestion |
| **Administrative and Social Consultations** |
| Absence of Urgent Psychiatric Psychopathology  Administrative Consultation  Administrative Discharge  Administrative Error  Admitted for Containment  Admitted for Evaluation  Asks for Prescriptions  Consultation  Instrumental Use of Medical Services  Involuntary Admission for Evaluation  Leaves the ED Before Being Attended  Leaves Without Being Assessed  Leaves Without Being Seen  Loss of Documents  Medication Adjustment  Medication Consultation  No Acude Psychiatric Pathology Observed  No Psychiatric Pathology  No Psychopatology  No Show  No Urgent Psychopathology  Not Seen  Prescriptions  Psychiatric Admission  Referred for Hospital Admission  Report  Requests a Report  Requests Medication  Requests Prescriptions  Requests Treatment Adjustment  Social Dystocia  Social Problems  Transfer to Another Hospital  Verification of Medication Regimen  Voluntary Discharge |
| **Behavioural Alterations Not Otherwise Specified** |
| Aggression  Aggressiveness  Aggressivity Crisis  Agitation  Altered Behaviour  Behaviour Alteration  Behavioural Alteration Under Study  Emotional and Behavioural Alteration  Heteroaggressiveness  Psychomotor Agitation  Remitted Behavioural Alteration  Under Study |
| **Other Diagnoses at Discharge** |
| Adolescence Crisis  Apathy  Behaviour Disorganization  Catatonia  Catatonic Disorder  Catatonic Symptoms  Catatonic Syndrome  Containment  Decompensation  Deferred Diagnosis  Discomfort  Dissocial Behaviour  Emotion and Behaviour Disorder  Emotion Disorder  Emotional Instability  Irregular Treatment Adherence  Low Mood Under Study  Medication Noncompliance  Mood Alteration  Mutism  Other Emotional and Behavioural Disorders  Pending Results  Possible Affective Disorder  Previous Diagnoses  Problem Related to Support Group  Psychiatric  Psychopathological Decompensation  Test  Unspecified Disorder  Z Code |

**eMethods S2**: Substances included under each category.

| **Substance categories** |
| --- |
| **Alcohol**  **Stimulants**: Amphetamine, Methamphetamine, Cocaine, Lisdexamfetamine, Speed  **Benzodiazepines and other hypnosedatives**: Benzodiazepine, Pregabalin  **Opioids**: Heroin, Opioids, Methadone  **Cannabinoids**: Cannabinoids, Marihuana, THC  **Unspecified / multiple substances** |

|  | **2019** | **2020** | **2021** | **2022** | **2023** |
| --- | --- | --- | --- | --- | --- |
| **January** | 244 | 303 | 243 | 322 | 335 |
| **February** | 245 | 299 | 229 | 323 | 311 |
| **March** | 277 | 220 | 272 | 340 | 354 |
| **April** | 255 | 350 | 242 | 270 | 309 |
| **May** | 253 | 287 | 237 | 329 | 338 |
| **June** | 286 | 248 | 307 | 336 | 332 |
| **July** | 245 | 228 | 290 | 281 | 331 |
| **August** | 274 | 226 | 238 | 325 | 365 |
| **September** | 291 | 234 | 305 | 317 | 388 |
| **October** | 294 | 256 | 309 | 343 | 376 |
| **November** | 263 | 216 | 298 | 313 | 305 |
| **December** | 250 | 213 | 313 | 339 |  |

**Table S1**. Number of monthly visit counts for psychiatric care patients across the study period. Pre-pandemic period is shadowed in yellow; pandemic period is shadowed in orange; post-pandemic period is shadowed in green.

|  | **2019** | **2020** | **2021** | **2022** | **2023** |
| --- | --- | --- | --- | --- | --- |
| **January** | 8355 | 9002 | 6425 | 7915 | 7881 |
| **February** | 7355 | 8132 | 5794 | 7143 | 7606 |
| **March** | 8311 | 5106 | 7066 | 8583 | 8470 |
| **April** | 8101 | 3621 | 7660 | 8360 | 7859 |
| **May** | 8349 | 5224 | 7754 | 8946 | 8294 |
| **June** | 8021 | 5781 | 8012 | 8498 | 7710 |
| **July** | 7981 | 6330 | 8087 | 8431 | 7916 |
| **August** | 7731 | 6043 | 7403 | 8005 | 8181 |
| **September** | 7959 | 6363 | 7997 | 8118 | 8178 |
| **October** | 8585 | 6441 | 8391 | 8888 | 8825 |
| **November** | 7873 | 6026 | 8026 | 7817 | 8107 |
| **December** | 8425 | 6139 | 8278 | 8227 |  |

**Table S2**. Number of monthly visit counts for medical and traumatological care patients across the study period. Pre-pandemic period is shadowed in yellow; pandemic period is shadowed in orange; post-pandemic period is shadowed in green.

| **Category** | **Pre-Pandemic %**  **(N/month)** | **Pandemic %**  **(N/month)** | **Post-Pandemic %**  **(N/month)** |
| --- | --- | --- | --- |
| Alcohol | 59.34%  (15.43) | 56.13%  (12.35) | 55.00%  (16.50) |
| Stimulants | 7.97%  (2.07) | 8.30%  (1.83) | 9.55%  (2.86) |
| Benzodiazepines and hypnosedatives | 3.30%  (0.86) | 3.75%  (0.83) | 3.94%  (1.18) |
| Opioids | 1.37%  (0.36) | 2.96%  (0.65) | 1.82%  (0.55) |
| Cannabinoids | 1.10%  (0.29) | 1.19%  (0.26) | 2.12%  (0.64) |
| Multiple substances and/or non-specified substances | 26.92%  (7.00) | 28.46%  (6.26) | 26.06%  (7.82) |

**Table S3**. Number of monthly visits and percentage of the monthly total per substance over the study period.
